# An Unusually Large Parakeratinised Odontogenic Keratocyst in the Maxilla With Extension Into the Floor of the Maxillary Sinus

**DOI:** 10.7759/cureus.21002

**Published:** 2022-01-07

**Authors:** Karthik Rajaram Mohan, Saramma mathew Fenn, Ravikumar Pethagounder Thangavelu, Jeyavel MJ, Durgadevi Pancharethinam

**Affiliations:** 1 Oral Medicine and Radiology, Vinayaka Missions Sankarachariyar Dental College, Salem, IND

**Keywords:** surgical enucleation, satellite cysts, odontogenic keratocyst, maxillary sinus, maxilla

## Abstract

Parakeratinised odontogenic keratocyst is a developmental odontogenic cyst affecting the jaw with aggressive behaviour, rapid growth, extension into adjacent structures, and a high recurrence rate of 4.4% in either jaw. This cyst usually occurs in the molar-ramus region of the mandible. If it occurs in the maxilla, the cyst can traverse through the trabecular bone marrow spaces resulting in expansion more mediolaterally than in the buccolingual direction. Here, we present you a case of peripherally occurring parakeratinised variant of odontogenic keratocyst in a 20-year-old-male in relation to impacted left maxillary cuspid in the maxilla with involvement of the floor of the maxillary sinus, imaged by cone-beam computed tomography systems (CBCT) and treated by surgical enucleation.

## Introduction

An odontogenic keratocyst is a developmental odontogenic cyst affecting the jaw, which is usually filled with keratin and has the most disputable pathologies affecting the maxillofacial region. Some thought such odontogenic keratocysts are derived from the remnants of dental lamina found in gingiva and periodontal ligament, after the development of tooth and possibility of offshoots of such remnants on the soft tissue located distal to the last molar, hence found to be more common in the molar-ramus area of the mandible. One of the main characteristics of such cysts is to grow along the cancellous channels, hence extending more mediolaterally than buccolingual direction causing very little or no bony expansion. Usually, patients affected by such odontogenic keratocysts can present with swelling resulting in facial asymmetry, associated with pain and abnormal discharge. Because of its tendency for recurrence and aggressive clinical behaviour, it was renamed as a keratocystic odontogenic tumour or a benign neoplasm occurring intraosseously by El-Nagger et al, World Health Organization classification of Head and Neck Tumours (2017) [1]. Nearly half of the odontogenic keratocyst usually occurs at the mandibular angle and extends forward into the body of the mandible. In the maxilla, it can extend into the floor of the maxillary sinus and nasopalatine region close to the midline [1]. The cyst is initially free of symptoms but becomes symptomatic when it invades the floor of the maxillary sinus or nose and the entire ascending ramus, involving the coronoid process [1]. The word "parakeratinised" refers to incomplete keratinization of the stratum corneum, which appears as flattened tonofilaments with nuclei and other organelles retained [1]. Parakeratinised epithelium 5-8 cells thick is a common histological characteristic [1]. The recurrent nature of the parakeratinised odontogenic cyst is due to its fragile lining, which results in incomplete removal of the cystic epithelial lining, the presence of daughter cysts in its wall or epithelial islands, and epithelial off-shoots, known as microcysts in the cyst's epithelial lining [1].

## Case presentation

A 20-year-old male patient reported to our oral medicine department with a chief complaint of pain and swelling in his left middle third of the face for the past two months. According to history, it began as a small swelling and gradually grew to its current size of approximately 3.5 x 4 cm. The swelling was associated with tenderness, which was mild, intermittent, and non-radiating in nature, non-pulsatile; the surface of the swelling is smooth, firm in consistency, fluctuant in nature. He visited a dentist nearby for the same problem of swelling on the left side of his face, for which Augmentin (Amoxicillin 500mg + Potassium clavulanate 125mg) twice daily for five days was prescribed to him. Even after taking the medication, the swelling did not subside. He had no history of similar swellings in other parts of his body. No malaise, discharge, nasal blockage, or paresthesia were associated with the swelling. The patient did not give any history of recent trauma on the left side of the face or the tooth. He also gives no history of deleterious oral habits like chewing or smoking forms of tobacco or alcohol consumption. He was afebrile. His pulse rate was 87 beats/min, and his blood pressure was 120/71 mm Hg. 

**Figure 1:**
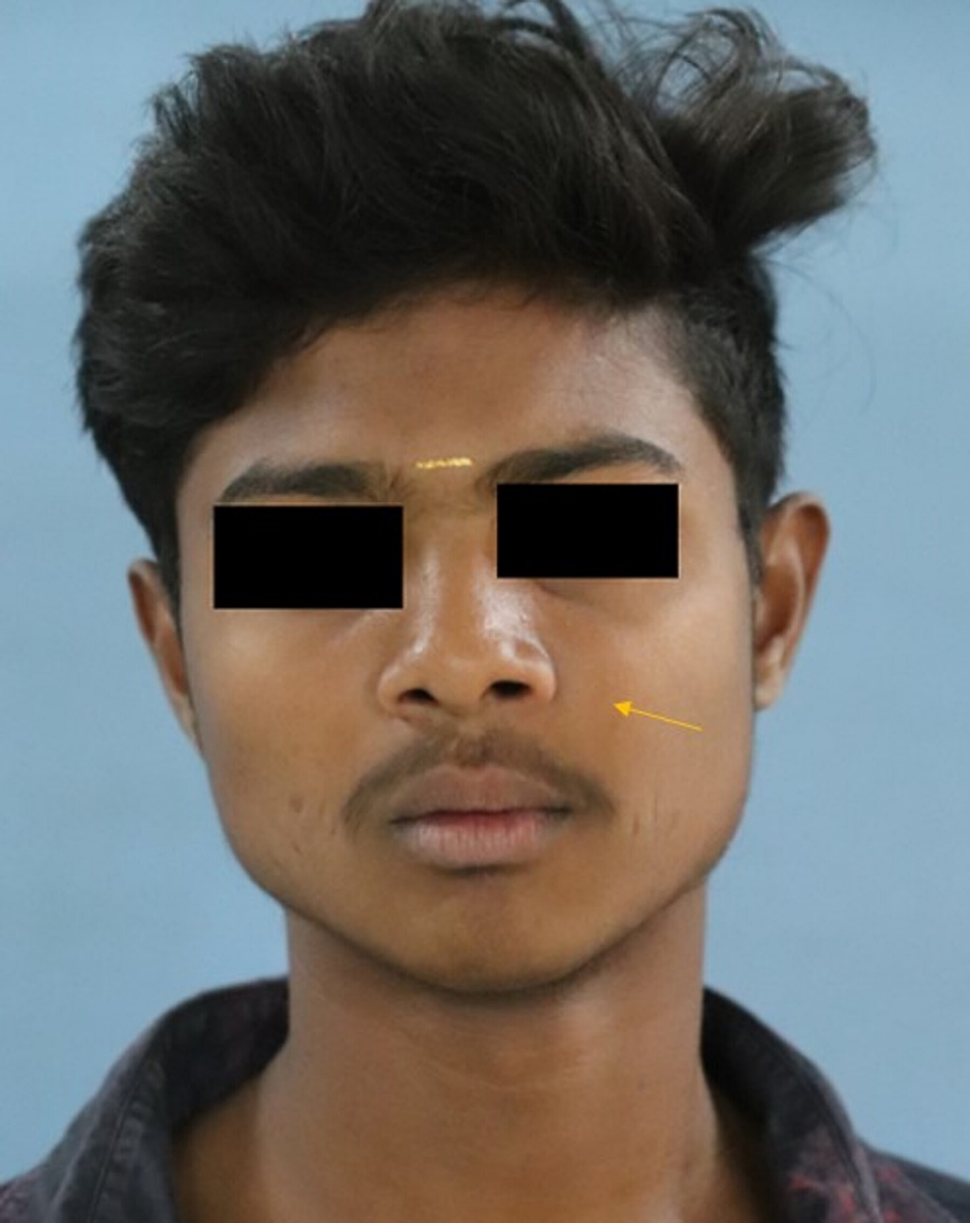
Extraoral examination revealed swelling on the left, middle third of the face obliterating the left nasolabial fold

Extraoral examination on inspection found facial symmetry present on the left side of the face due to the presence of a diffuse swelling on the left middle third of the face, measuring about 2 x 4 cm in size, extending 2 cm away from the left ala of the nose. Superoinferiorly, it extended 2 cm below the left ala-tragal plane and inferiorly, 1 cm above the corner near the left vermilion border of the lip. The nasolabial fold is completely obliterated due to the presence of swelling on the middle-third on the left side of the face. Absence of a pin-point opening or extraoral sinus on the surface of the swelling (Figure 1). The skin over the surface of the swelling was stretched and not shiny. No visible colour changes were noted when compared to the adjacent skin on the surface of the swelling. A single palpable left submandibular lymph node was present, measuring approximately 0.5 cm in diameter, firm in consistency, tender on palpation, and mobile. 

On palpation, all the inspection findings regarding the site, size, shape, surface, and extent were confirmed on palpation. The extraoral swelling was firm in consistency and non-compressible, non-reducible. The skin over the surface of the swelling was pinchable. Mild tenderness was present only along the inferior border of the swelling. Intraoral examination revealed a single, oval-shaped intraoral swelling involving the buccal aspect of the attached gingiva and obliteration of the left maxillary buccal vestibule from the 23 to 25 teeth region. It was approximately 3 x 4 cm in size, extending anteriorly from the attached gingiva of 23 posteriorly to the distal of the attached gingiva in relation to 25, superior extent cannot be specified, and inferiorly to the marginal gingiva in relation to 23, 24, 25 teeth region. The left maxillary buccal vestibule from 23 to 25 is obliterated.

**Figure 2:**
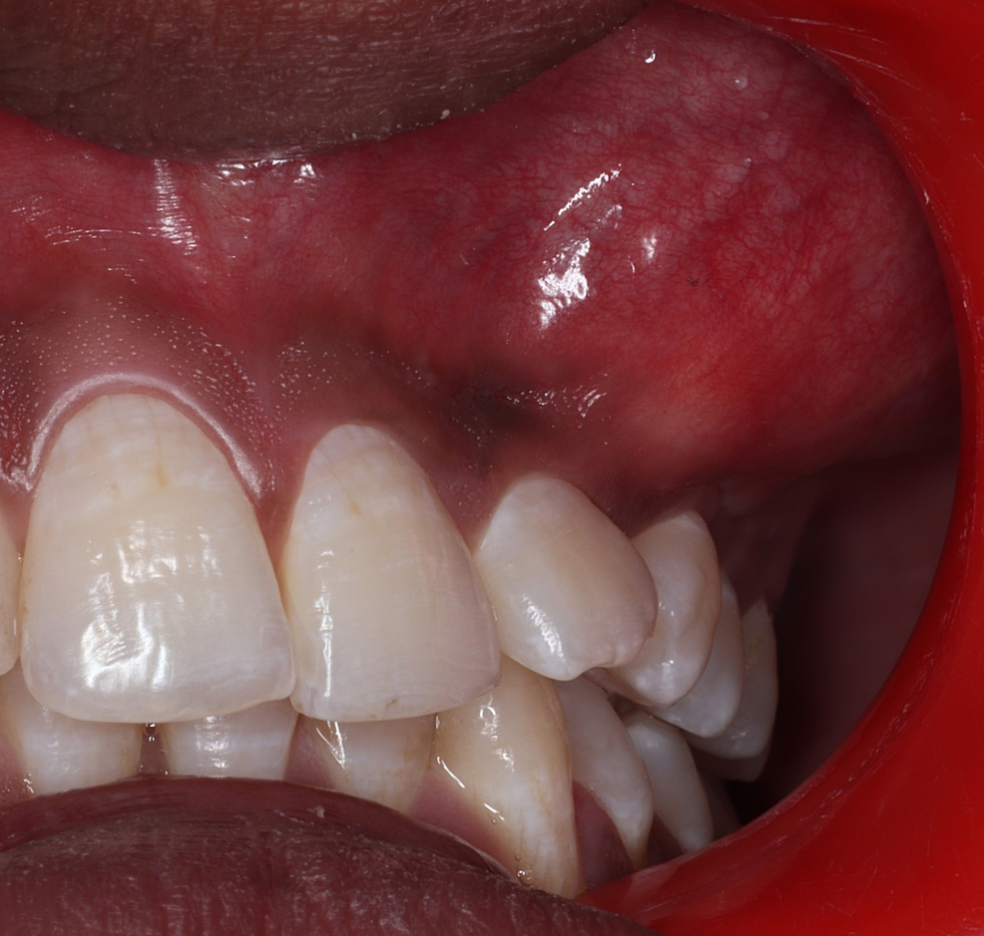
Intraoral examination revealed a swelling resulting in obliteration of left mucobuccal vestibule in relation to 23, 24, 25 region

The margins were well defined inferiorly and diffused superiorly. The mucosa of the swelling was smooth, normal in colour to the adjacent mucosa, with no prominence of blood vessels or engorged veins and no visible pulsation. No pus discharge or bleeding was present. Tooth 22 was displaced distally, and 24 was displaced medially. Twenty-three was clinically absent (Figure 2). On palpation, all inspection findings regarding site, size, and shape were confirmed. It had mixed consistencies of bony hard at the superior extent of the swelling and soft consistency at the inferior extent of the swelling, respectively. The swelling was fluctuant at the inferior part and was non-compressible, non-reducible, and non-tender on palpation. Eggshell cracking was present on palpating the inferior part of the swelling. The differential diagnoses considered were dentigerous cyst, nasolabial cyst, radicular cyst, odontogenic keratocyst, adenomatoid odontogenic tumour, and ameloblastic fibroma. The various investigation modalities advised were intraoral periapical radiograph, topographic maxillary occlusal radiograph, cone-beam computed tomography systems (CBCT), and pulp vitality test by electric pulp tester. There was no response from 24 by the electric pulp testing method. The left maxillary second premolar 25 revealed a delayed response to electric pulp testing at a value of 18.

**Figure 3:**
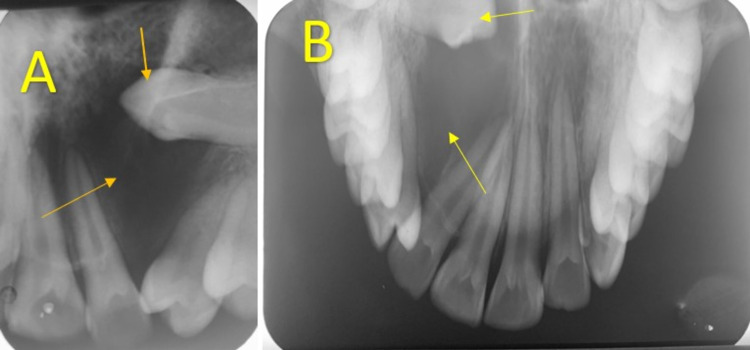
Intraoral periapical radiograph 3A. Intraoral periapical radiograph revealed a well-defined radiolucency greater than 1.5 cm in diameter encircling the crown of 23; 3B. Topographic maxillary occlusal radiograph revealed a well-defined radiolucency greater than 1.5 cm in diameter encircling the crown of impacted 23 and pathological migration with an  increased distal inclination of root of 22.

An intraoral periapical radiograph (IOPA) revealed a well-defined radiolucency measuring more than 1.5 cm in diameter in relation to impacted 23. The well-defined radiolucency bordered by a radiopaque sclerotic border was seen to be slightly apical to the cementoenamel junction. The lamina dura was lost in 21 but intact in 22. There was a severe axial inclination of the crown of 24, too (Figure 3A). The topographic maxillary occlusal radiograph revealed a well-defined radiolucency bordered by a radiopaque sclerotic border attached near the cementoenamel junction of impacted 23. The radiographic contrast of impacted 23 appeared more radiodense than the adjacent tooth contrast, suggesting an impacted 23 (Figure 3B).

**Figure 4:**
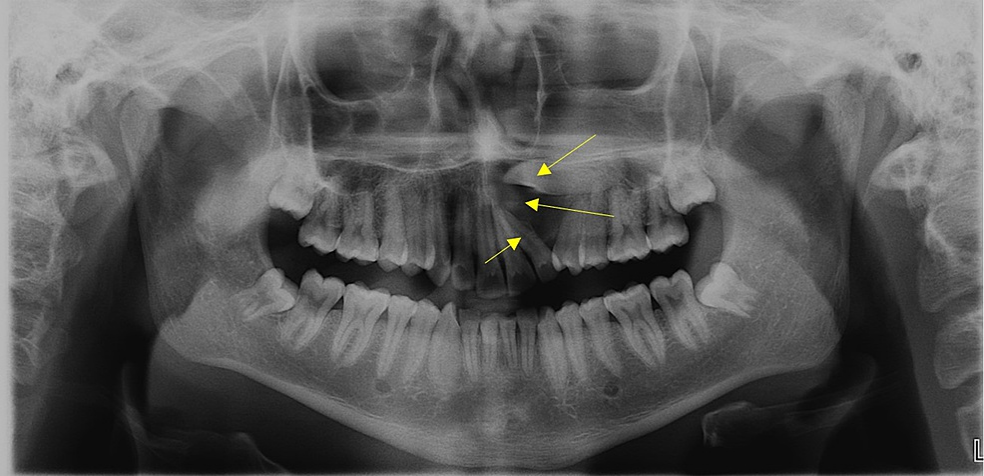
Digital orthopantomograph It revealed a well-defined radiolucency in relation to impacted left permanent maxillary canine 23 and pathological migrated with an increased distal inclination of root of 22

Digital panoramic tomography revealed an impacted 23 and the presence of a well-defined radiolucency greater than 1.5 cm in diameter around the impacted 23 (Figure 4).

**Figure 5:**
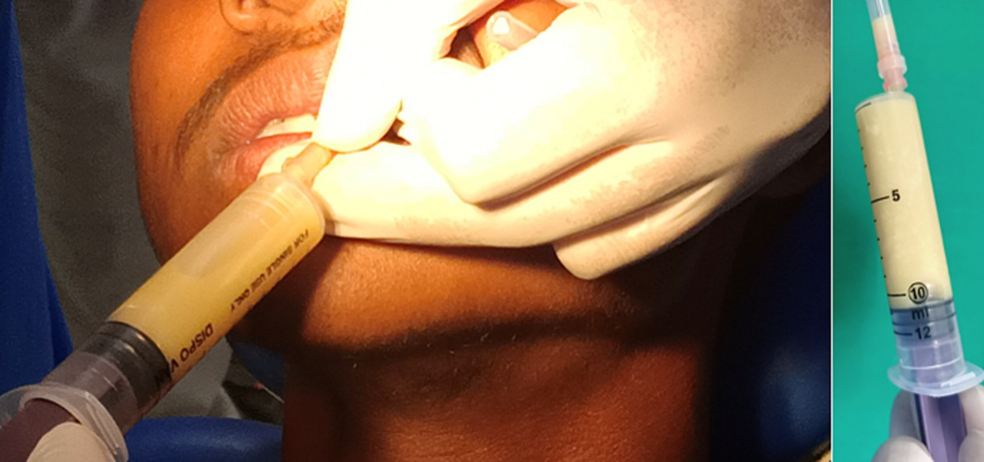
Aspiration revealed a high viscous white creamy fluid

A 15-gauge disposable syringe with a wide bore needle was used for aspiration. A highly viscous white, creamy fluid was obtained by aspiration (Figure 5). The aspirated fluid was sent for analysis, which revealed abundant keratin. The size of the swelling was reduced to 1 x 2 cm after aspiration.

**Figure 6:**
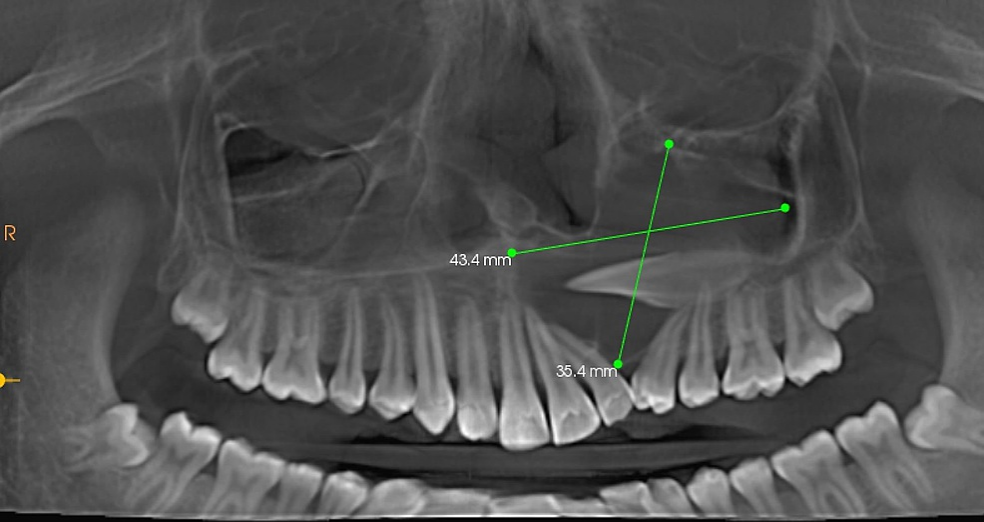
CBCT Panoramic mode revealed a well-defined radiolucency measuring 43.4 mm x 30.4 mm around impacted 23.

The panoramic mode by CBCT imaging revealed a homogenous mass measuring about 43.4 mm x 35.4 mm extending mediolaterally from the midline of 21 to 26, craniocaudal from the roof of the left maxillary sinus wall to the floor of the sinus, and inter-radicular between displaced 22 and 24. The teeth 22 and 24 were displaced, and there was a change in their axial inclination. There was no root resorption or calcification within the homogenous expansile mass (Figure 6).

**Figure 7:**
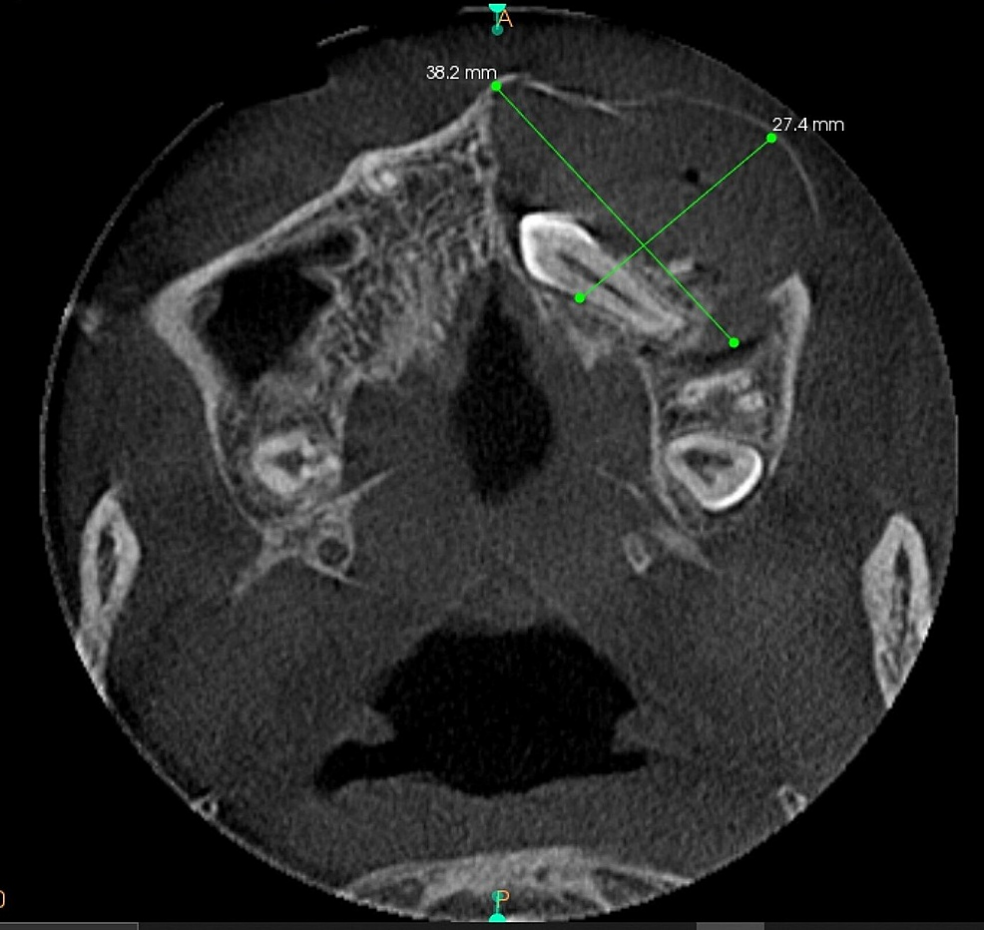
The axial section of CBCT It revealed a single radiopaque cystic lesion seen on the left side with buccal expansion and not crossing the midline around a horizontally impacted 23. Loculation is seen on the buccal periphery with a buccal cortical breach seen on the posterior side of the lesion.

The axial section of the CBCT at the level of the maxillary sinus revealed a single irregularly shaped isodense area within the left maxillary sinus of about 27.4 mm x 38.2 mm, and a pathologically migrated tooth-like radiopaque crown-like structure resembling the morphology of left maxillary canine, within the left maxillary alveolar bone. The walls of the maxillary sinus were intact. There was no variation in the size of the right and left maxillary air sinuses (Figure 7).

**Figure 8:**
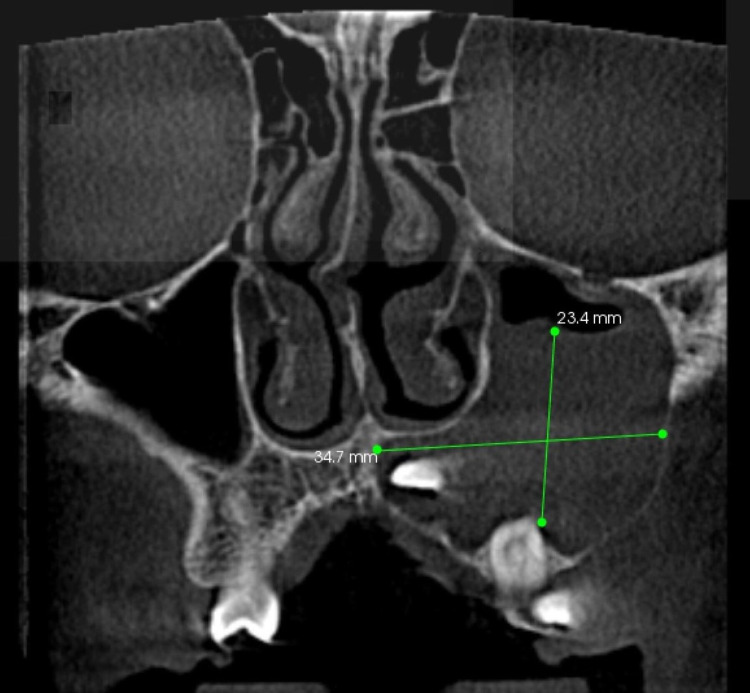
The coronal section of the CT image The CT image at the level of the maxillary left first premolar revealed a well-defined, homogenous, irregularly shaped mass 54.7 mm x 23.4 mm seen involving the left maxillary sinus and surrounding the impacted 23 with buccal cortical expansion, and thinning of the Buccal cortical border is seen but intact

The coronal section of the CT image at the level of the maxillary left first premolar revealed a well-defined, homogenous, irregularly shaped radiopaque mass 54.7 mm x 23.4 mm seen involving the left maxillary sinus and surrounding the impacted 23 with buccal cortical expansion and thinning of the Buccal cortical border is seen but intact (Figure 8).

**Figure 9:**
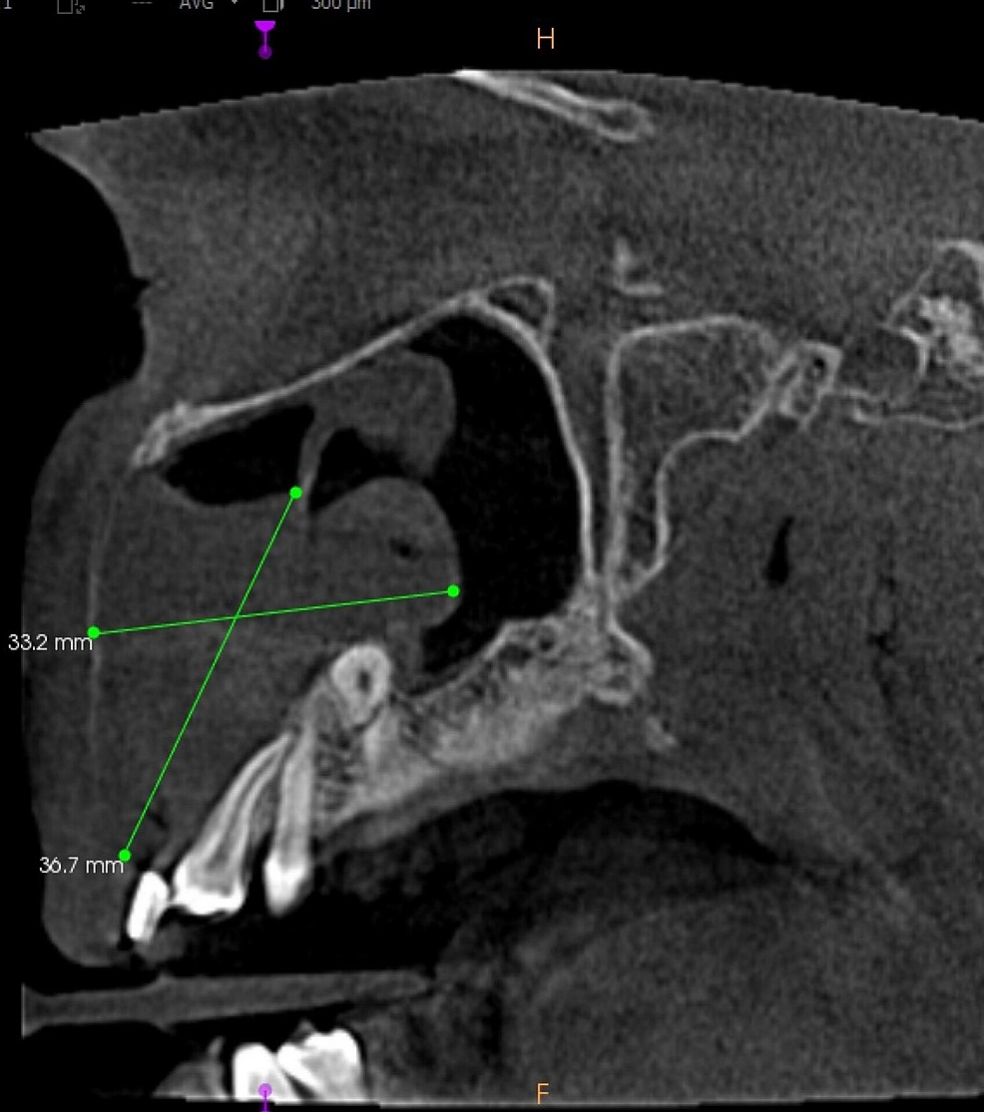
The sagittal section of CBCT revealed a homogeneous radiopaque mass measuring approximately 36.7 mm x 33.2 mm and extending buccally with a breach in the buccal cortical plate.

The sagittal section of CBCT revealed a homogeneous radiopaque mass measuring approximately 36.7 mm x 33.2 mm and extending buccally with a breach in the buccal cortical plate (Figure 9).

**Figure 10:**
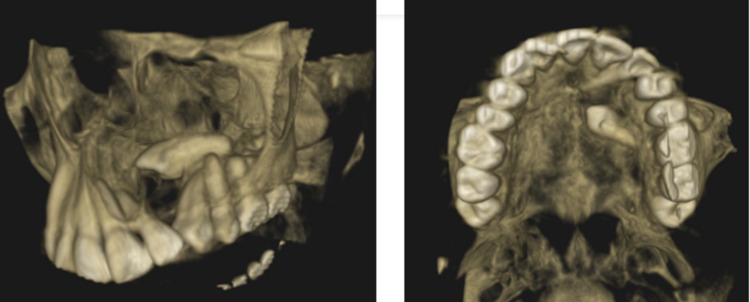
3D reconstructed CBCT revealed both buccal and palatal cortical perforation with palatally placed impacted left maxillary canine

The 3D-reconstructed CBCT image revealed both buccal and palatal cortical perforation with a palatally impacted left maxillary canine (Figure 10).

**Figure 11:**
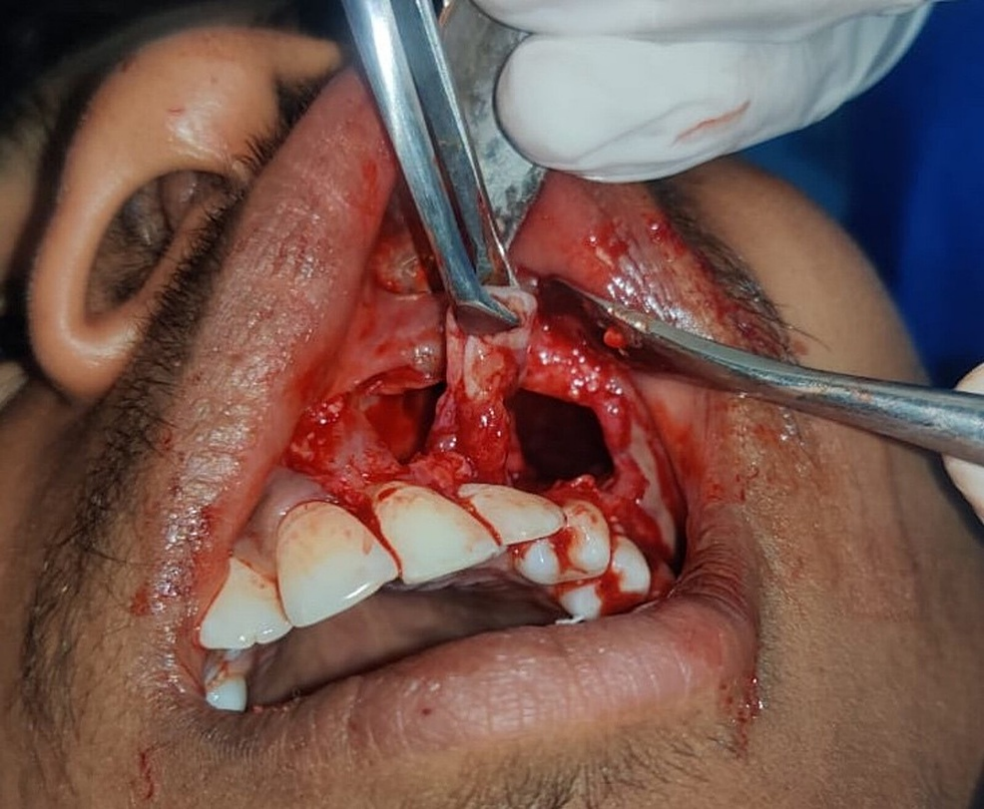
Mucoperiosteal flap reflected and cystic lining enucleated

**Figure 12:**
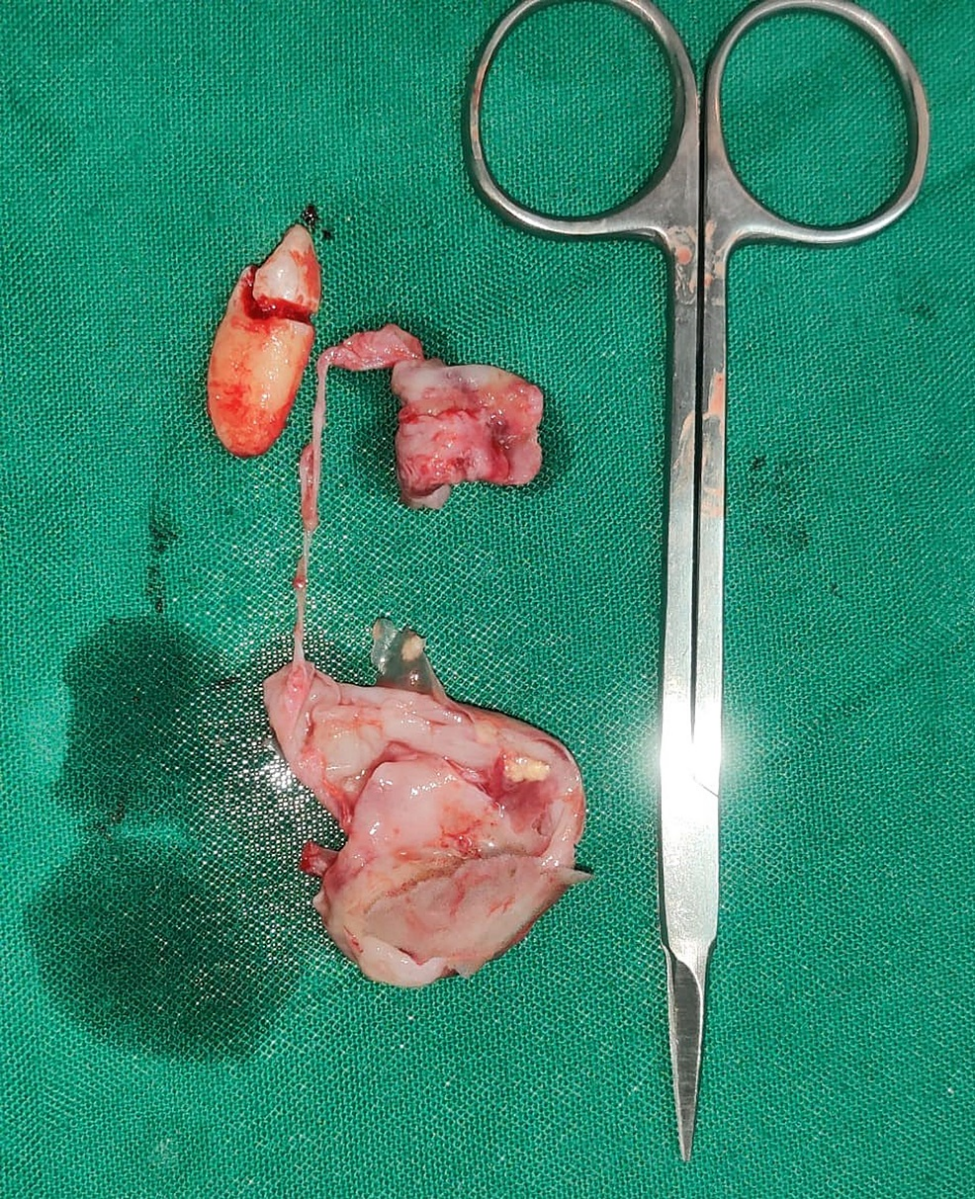
Enucleated cyst wall and impacted tooth

Under general anesthesia, the mucoperiosteal flap was reflected, and the cyst wall was surgically enucleated and sent for histopathological examination (Figure 11). The excised cystic wall and affected impacted tooth were removed (Figure 12).

**Figure 13:**
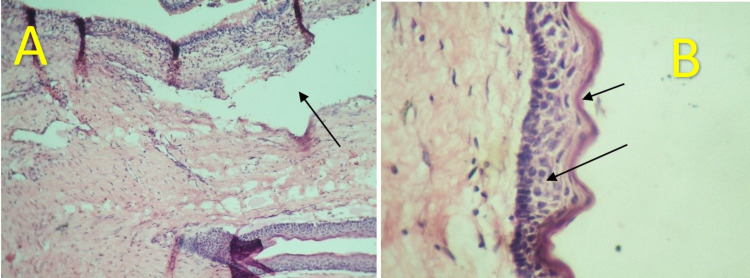
A. Histopathological low power photomicrograph (5x) revealed a cystic lumen lining, which is uniform in thickness B. High power photomicrograph (10x) revealed a corrugated parakeratinised stratified squamous epithelium with basal cell palisading

The histopathological photomicrograph revealed a cystic lumen lining, which was uniform in thickness with corrugated parakeratinised stratified squamous epithelium with basal cell palisading. The epithelium was separated from the underlying connective tissue, infiltrated by chronic inflammatory cells. Daughter cells were discovered in several connective tissue locations, as well as portions of the epithelium lining the maxillary sinus and blood vessels (Figures 13A, 13B).

**Figure 14:**
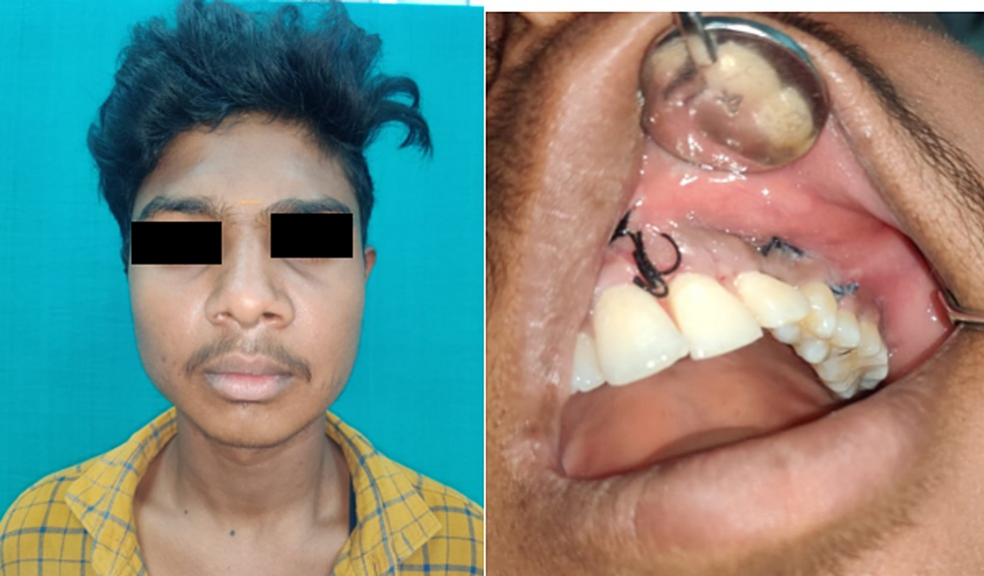
Postoperative extraoral and intraoral clinical photograph

The final diagnosis of peripheral parakeratinised odontogenic keratocyst in relation to impacted canine 23 extending into the floor of the maxillary sinus was made after histopathological examination. The patient was followed up after six months. Healing was satisfactory, and no recurrence was reported after six months (Figure 14).

## Discussion

The odontogenic keratocyst was first described by Philipsen in 1956 [1]. The histological criteria to diagnose odontogenic keratocysts were first published by Pindborg and Hansen in 1962. It was first thought to be a "Primordial cyst", as the origin of the lesion was found to be the tooth primordium. Shear described it as "keratocystoma". In 2004, Reichart and Philipsen described it as a "keratinising cystic odontogenic tumour." In 2005, Philipsen described it as a "keratocystic odontogenic tumour" [2].

Etiopathogenesis

Genetic Predisposition

The protein patched homolog (PTCH) gene mapped to chromosome 9q22.3 plays an important role in the development of odontogenic keratocyst. Some consider it to be a developmental odontogenic cyst that arises from the dental lamina. The mean age of occurrence is 10-40 years, with a male predominance. The posterior sextants of the jaws were commonly affected, involving the posterior body and ascending ramus. 25%-40% of the cases were associated with unerupted teeth [3,4]. Philipsen (2005) stated that even though the lesions appear smaller in the maxilla, they are extensive due to the cancellous nature of the maxillary bone [5]. Jansisyonant (2006) stated that the adjacent teeth are usually displaced or pathologically migrated, but resorption of the roots least likely occurs [6]. Brannon reported that the recurrence of parakeratinised odontogenic keratocyst is related to the surgical difficulty encountered in complete removal of the lesion. Our study was contradictory to the clinical findings of Wright, who stated that orthokeratinised odontogenic cysts were more commonly found in relation to the dentigerous cyst around an impacted tooth. The clinical finding in our case was buccal expansion, which was similar to the study by Kim (2009). Large lesions can cause thinning and perforation of the cortical plate [7].

Radiographic features

Radiographic differentiation of odontogenic keratocyst from other cysts remains difficult, and such cysts can be confused with a dentigerous cyst, residual cyst, lateral periodontal cyst, or nasopalatine cyst. The multilocular appearance can be confused with ameloblastoma. Hence, microscopic findings remain a gold standard in the diagnosis of parakeratinised odontogenic keratocyst. Histologically, it can resemble keratoameloblastoma, where separation and edema between the rest of the epithelium and the basal layer are seen. The differential diagnosis of peripheral parakeratinised odontogenic keratocyst includes dentigerous cyst, in which the radiopaque sclerotic border starts near the cementoenamel junction of the impacted tooth, whereas in parakeratinised odontogenic keratocyst, the radio-opaque sclerotic border is seen attached slightly apical to the cementoenamel junction. Aneurysmal bone cysts appear radiographically as scalloping between the tooth roots, whereas parakeratinised odontogenic keratocysts do not scallop between the roots of the affected tooth. In keratoameloblastoma, there will be marked cortical expansion, whereas, in parakeratinised odontogenic keratocyst, cortical expansion is not marked or minimal. The histological criteria for the diagnosis of parakeratinised odontogenic keratocyst were put forward by Pindborg and Philipsen and Henriksen in 1962. The presence of daughter cysts and cholesterol crystals in the fibrous connective tissue wall. Wysocki and Sapp stated that corrugated hypercellular epithelial lining and polarised basal epithelial lining were seen in parakeratinised odontogenic keratocyst [8].

Immunohistochemistry

Immunohistochemical staining with p63 was found to be positive for staining in the suprabasal epithelial lining of odontogenic keratocysts. The role of p63 in inhibition of growth and anti-apoptosis favours the proliferative potential of the epithelial lining and the aggressive behaviour of odontogenic keratocyst [9-12]. The expression of MMP-1 is associated with the degradation of the organic bone matrix. MMP-2 in the basement membrane of the epithelial lining degrades the extracellular matrix surrounding the odontogenic keratocyst, thus favouring its dissemination through trabecular bone marrow spaces [13]. Odontogenic keratocysts have higher levels of vascular endothelium growth factors [14]. Surgical enucleation of the cyst, peripheral ostectomy or surgical resection after enucleation, decompression with a non-inflatable tube, cryotherapy, and 1% Carnoys solution after surgical enucleation are the treatment modalities for peripheral parakeratinised odontogenic keratocyst based on its location and relationship to anatomical landmarks and vital structures. The use of non-chloroform-containing modified Carnoys solution reduced the recurrence of parakeratinised odontogenic keratocysts by 1.6% [15,16].

Complications

The parakeratinised odontogenic keratocyst can cause cortical expansion, resulting in cortical thinning, perforation, and leading to pathological fracture, if left untreated over a while. About 1% of such odontogenic keratocysts also tend to transform into ameloblastoma or squamous cell carcinoma [17-20].

## Conclusions

Parakeratinised odontogenic keratocyst is asymptomatic during the initial stages and gradually patients affected by such cysts, report with discomfort due to pain or swelling resulting in facial asymmetry. The pressure effects caused by such cysts on the adjacent vital structures, such as tooth can lead to pathological migration of teeth and loss of pulp vitality or the floor of the maxillary sinus can be involved mimicking symptoms of chronic maxillary sinusitis. A thorough clinical examination, as well as careful radiological and histopathological evaluation, aid in the diagnosis of parakeratinised odontogenic keratocyst, which, if left untreated, would have caused pathological fracture or transformed over a period of time into ameloblastoma or squamous cell carcinoma. Therefore, by intervention and prompt treatment of such parakeratinised odontogenic keratocyst, the adverse effects of pathological fracture or its malignant transformation is prevented and, thereby, the quality of life is maintained.

## References

[1] (2017). WHO Classification of Head and Neck Tumours. https://publications.iarc.fr/Book-And-Report-Series/Who-Classification-Of-Tumours/WHO-Classification-Of-Head-And-Neck-Tumours-2017.

[2] Shear M (2003). Odontogenic keratocysts: natural history and immunohistochemistry. Oral Maxillofac Surg Clin North Am.

[3] (2006). Pathology and Genetics of Head and Neck Tumours. Pathology and Genetics of Head and Neck Tumors.

[4] Augustine D, Rao RS, Lakshminarayana S, Prasad K, Patil S (2021). Sub-epithelial hyalinization, incomplete cystic lining, and corrugated surface could be a predictor of recurrence in odontogenic Keratocysts. J Oral Biol Craniofac Res.

[5] Shear M (2003). Odontogenic keratocysts: clinical features. Oral Maxillofac Surg Clin North Am.

[6] Crowley TE, Kaugars GE, Gunsolley JC (1992). Odontogenic keratocysts: a clinical and histologic comparison of the parakeratin and orthokeratin variants. J Oral Maxillofac Surg.

[7] Chirapathomsakul D, Sastravaha P, Jansisyanont P (2006). A review of odontogenic keratocysts and the behavior of recurrences. Ora Surg Ora Med Ora Pathol Ora Radio Endo.

[8] Wright JM (1981). The odontogenic keratocyst: orthokeratinized variant. Ora Surg Ora Med Ora Patho.

[9] Wysocki GP. Sapp JP (1975). Scanning and transmission electron microscopy of odontogenic keratocysts. Ora Surg Ora Med Ora Patho.

[10] Gurgel CA, Ramos EA, Azevedo RA, Sarmento VA, da Silva Carvalho AM, dos Santos JN (2008). Expression of Ki-67, p53 and p63 proteins in keratocyst odontogenic tumours: an immunohistochemical study. J Mol Histol.

[11] Kolár Z, Geierová M, Bouchal J, Pazdera J, Zboril V, Tvrdý P (2006). Immunohistochemical analysis of the biological potential of odontogenic keratocysts. J Oral Pathol Med.

[12] Agaram NP, Collins BM, Barnes L (2004). Molecular analysis to demonstrate that odontogenic keratocysts are neoplastic. Arch Pathol Lab Med.

[13] Eryilmaz T, Ozmen S, Findikcioglu K, Kandal S, Aral M (2009). Odontogenic keratocyst: an unusual location and review of the literature. Ann Plast Surg.

[14] Cavalcante RB, Pereira KM, Nonaka CF, Nogueira RL, de Souza LB (2008). Immunohistochemical expression of MMPs 1, 7, and 26 in syndrome and nonsyndrome odontogenic keratocysts. Ora Surg Ora Med Ora Patho Ora Radio Endo.

[15] Mitrou GK, Tosias KI, Kyroudi A, Sklavounou A (2009). Odontogenic keratocyst expresses vascular endothelial growth factor: an immunohistochemical study. J Oral Pathol Med.

[16] Donnelly LA, Simmons TH, Blitstein BJ (2021). Modified Carnoy's compared to Carnoy's solution is equally effective in preventing recurrence of odontogenic keratocysts. J Oral Maxillofac Surg.

[17] Preston RD, Narayana N (2005). Peripheral odontogenic keratocyst. J Periodontol.

[18] Stoelinga PJ (2003). Excision of the overlying, attached mucosa, in conjunction with cyst enucleation and treatment of the bony defect with carnoy solution. Oral Maxillofac Surg Clin North Am.

[19] Blanas N, Freund B, Schwartz M, Furst IM (2000). Systematic review of the treatment and prognosis of the odontogenic keratocyst. Ora Surg Ora Med Ora Patho Ora Radio Endo.

[20] Maria A, Sharma Y, Chhabria A (2011). Squamous cell carcinoma in a maxillary odontogenic keratocyst: a rare entity. Natl J Maxillofac Surg.

